# A novel human *Streptomyces* strain redresses malignancy by inducing apoptosis and regaining gut homeostasis

**DOI:** 10.1128/msystems.00528-26

**Published:** 2026-09-23

**Authors:** Yu-Hui Wang, Bao-Juan Yuan, Yao Meng, Hong-Tao Xu, Xin-Yu Gao, Liang Liu, Hong-Da Tian, Xiaoyu Wang, Xing-Hua Zhang, Song-Ling Zhu, Randal N. Johnston, Le Tang, Gui-Rong Liu, Shu-Lin Liu

**Affiliations:** 1 Genomics Research Center (Key Laboratory of Gut Microbiota and Pharmacogenomics of Heilongjiang Province, State-Province Key Laboratory of Biomedicine-Pharmaceutics of China), College of Pharmacy, Harbin Medical University 34707, Harbin, China; 2 Harbin Medical University-University of Calgary Cumming School of Medicine Centre for Infection and Genomics, Harbin Medical University 34707, Harbin, China; 3 State Key Laboratory of Frigid Zone Cardiovascular Diseases, Harbin Medical University 34707, Harbin, China; 4 Department of Pathogen Biology, Shantou University Medical College, Shantou, Guangdong, China; 5 Department of Biochemistry and Molecular Biology, University of Calgary 192289, Calgary, Canada; 6 Department of Microbiology, Immunology and Infectious Diseases, University of Calgary 198998, Calgary, Canada; Vanderbilt University Medical Center, Nashville, Tennessee, USA

**Keywords:** colorectal cancer, *Streptomyces*, homeostasis, gut ecology, natural species of bacteria

## Abstract

Colorectal cancer is among the deadliest malignancies globally. The modern therapies do not lead to ideal outcomes due mostly to drug resistance and recurrence. Previously, we treated cancer patients by ameliorating the gut microbiome and achieved curative effects. We hypothesize that anticancer bacteria might be ubiquitous in the gut but suppressed under certain conditions, pointing to a possibility that an ameliorated gut ecology may help regain the abundance or functions of the hypothetical anticancer bacteria. Here, we show our successful isolation and polyphasic characterization of a gut bacterial strain, CGMCC 17111, which exhibited potent suppressive effects on colorectal cancer. CGMCC 17111 belongs to *Streptomyces* and is most closely related to the type strain of *Streptomyces albidoflavus*, DSM 40455^T^. However, DSM 40455^T^ does not show any appreciable anticancer effects. Genetic, phenotypic, and functional analyses established CGMCC 17111 as representing a novel gut bacterial species with peculiar activities to redress malignancy without eliciting violent inflammatory reactions in the host.

**IMPORTANCE:**

The human gut bacterial strain *Streptomyces* sp. CGMCC 17111 represents a genetically distinct gut-derived *Streptomyces* lineage that exhibited antitumor activity in both *in vitro* and *in vivo* experimental models. As a resident member of the human gut microbiota, CGMCC 17111 may contribute to host–microbiota interactions that influence tumor-associated biological processes. The findings of this study provide a foundation for future research into gut-derived microbial products and their potential applications in cancer therapy while preserving host physiological homeostasis.

## INTRODUCTION

Colorectal cancer (CRC) remains a devastating malignancy with poor curability (1). The high mortality of CRC stems from insidious onset leading to late diagnosis, aggressive growth, and widespread metastasis, imposing a substantial burden on global population health (2). Current treatments, such as chemotherapy and immunotherapy, do not always lead to ideal outcomes, and their clinical use is often limited by severe side effects, variable patient responses, and resistance (3, 4). Given these challenges, novel therapeutic strategies are urgently needed. Over the past decades, microbial therapies, such as those utilizing bacterial metabolites or engineered probiotics, have emerged as promising alternatives due to their appreciable effects and reduced toxicity (5). However, curative outcomes by such products remain yet to be achieved due mostly to the instability of many of the bacterial products or rapid clearance of the probiotics by the host microbiota.

Close associations of gut dysbiosis with cancer have continually been reported (6). Recent findings have led to the postulation that dysbiosis may perturb the composition and functions of the gut microbiome, leading to the suppression of resident anticancer bacteria. For example, CRC patients have significantly lower diversity and abundance of *Escherichia coli* (7), suggesting that some lineages of *E. coli* might be protective against cancer and further pointing out the possibility that anticancer bacteria of diverse phylogenetic origins may exist in the gut. Based on this postulation, we screened the human gut bacteria and isolated Actinomycetota and Bacillota strains that have potent anticancer activities (8). Among these bacteria is one strain, CGMCC 17111, which could effectively halt CRC progression and achieve curative outcomes in a mouse model by ameliorating the gut microbiome, coordinating with immunity, and inducing cancer cell apoptosis.

In this study, we conducted systematic genomic and phenotypic characterization of CGMCC 17111. We found that CGMCC 17111 is most closely related to the type strain of *Streptomyces albidoflavus*, DSM 40455^T^, sharing 99.72% 16S rDNA similarity with it. However, we did not find any appreciable anticancer activities in *S. albidoflavus* DSM 40455^T^, suggesting their biological or even phylogenetic divergence. Based on the 3Cs criteria for delineating closely related bacteria into discrete natural species (9–11), we demonstrate that CGMCC 17111 represents a novel natural species of the genus *Streptomyces*, with its potent anticancer activity as the most distinctive biological trait compared to other *Streptomyces* lineages.

## RESULTS

### CGMCC 17111 is most closely related to *S. albidoflavus* DSM 40455^T^

**Fig 1:**
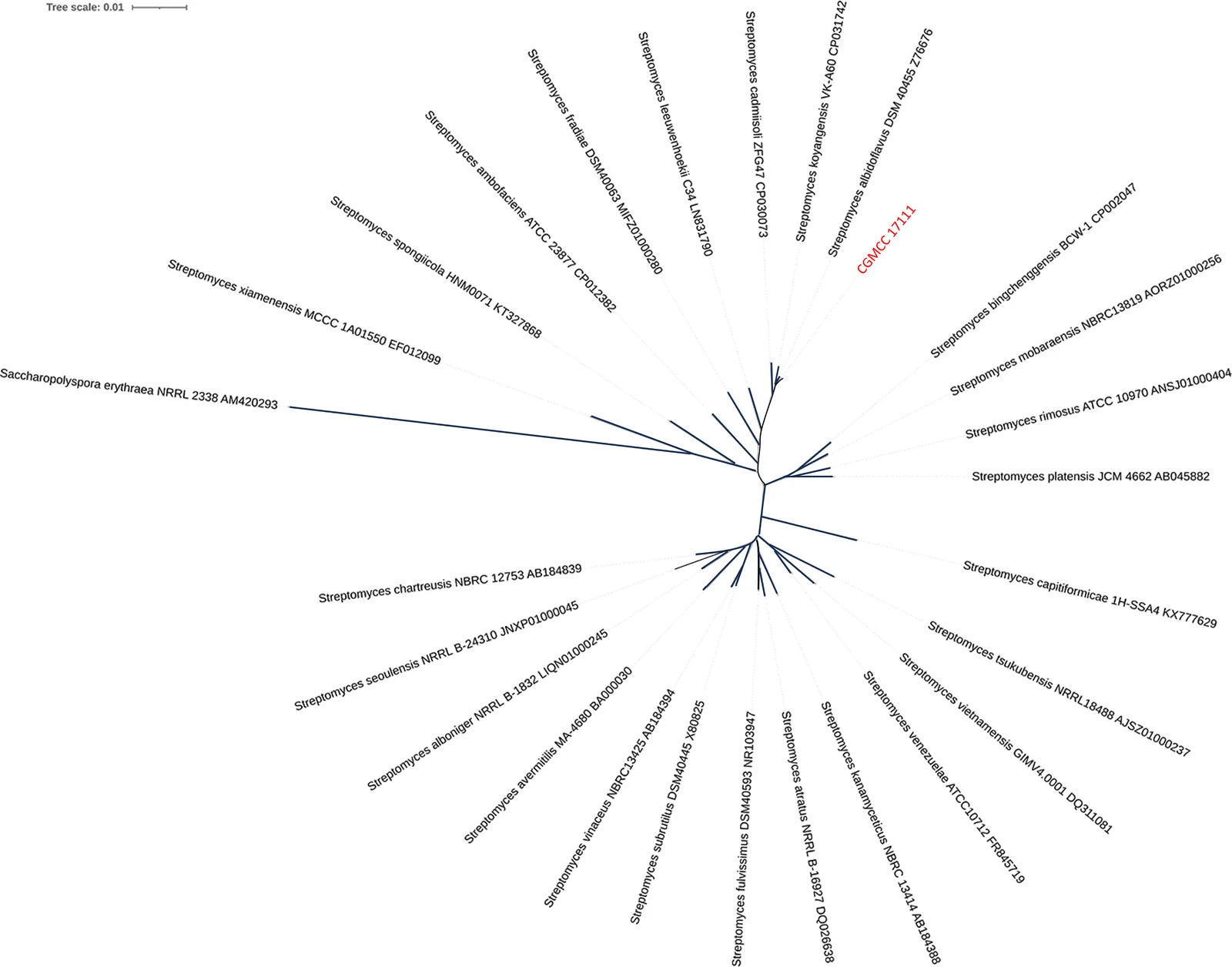
Neighbor-joining tree of CGMCC 17111 with *Streptomyces* type strains based on 16S rRNA gene sequences. Bootstrap values were based on 1,000 replicates. Bar, 0.01 substitutions per nucleotide position. *Saccharopolyspora erythraea* NRRL4338^T^ was used as outgroup.

Comparison of 16S rRNA gene sequences assigned the anticancer strain CGMCC 17111 to the genus *Streptomyces*, most closely related to *S. albidoflavus* DSM 40455^T^ (Fig. 1). Whole-genome comparisons of CGMCC 17111 with *S. albidoflavus* DSM 40455^T^ showed a DNA–DNA hybridization (DDH, ≥70% set for bacteria of the same taxonomic species) value of 75.20% and an average nucleotide identity (ANI; ca. 95%–96% being considered equivalent to a DDH value of 70%) value of 95.95%, which are both consistent with the 16S rDNA comparison result (99.72% shared by the two strains) between them, sufficient to recognize CGMCC 17111 as a member of the taxonomic species *S. albidoflavus*.

### Potent anticancer activities in CGMCC 17111 but not in *S. albidoflavus* DSM 40455^T^

**Fig 2:**
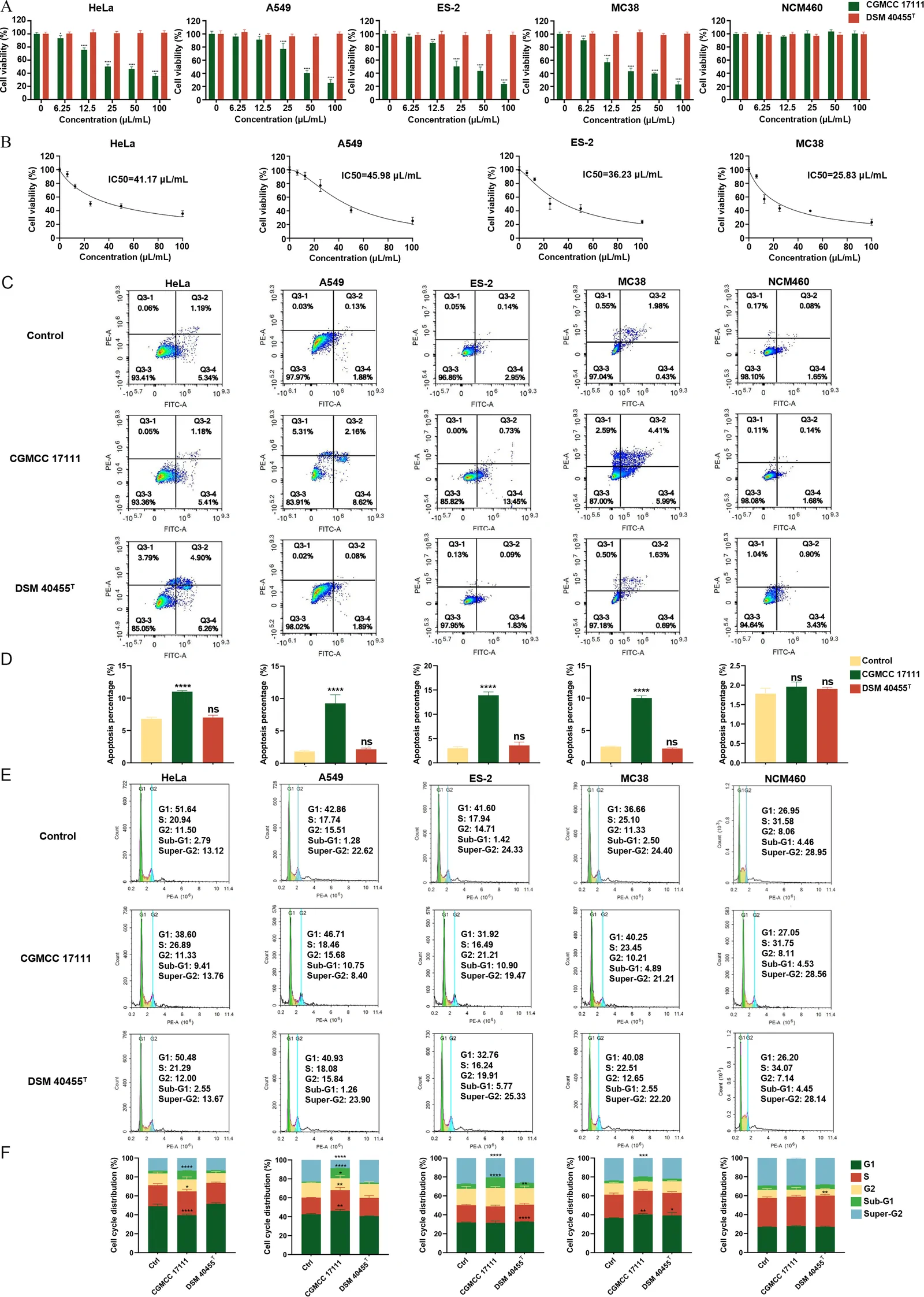
Suppressive effects of CGMCC 17111 on cancer cell lines in comparison with *S. albidoflavus* DSM 40455^T^. (**A**) Dose dependence of anti-cancer effects of CGMCC 17111 compared with DSM 40455^T^ by CCK8 assay. (**B**) IC50 determination of CGMCC 17111 culture supernatant on cancer cell viability. (**C**) Apoptosis induced by CGMCC 17111 compared with DSM 40455^T^ detected by flow cytometry. (**D**) Quantitative analysis of apoptotic cells, total apoptotic rate = Q3-2 + Q3-4. (**E**) Flow cytometry results to analyze the cell cycle distribution of GC cells treated with CGMCC 17111 compared with DSM 40455^T^. (**F**) The cell cycle results. Data were presented as mean ± standard deviation (SD) of at least three independent experiments. ns, *P* > 0.05; *P < 0.05, **P ≤ 0.01, ***P ≤ 0.001, ****P < 0.0001 vs control by one-way ANOVA (**D**) or by two-way ANOVA (**F**).

However, CGMCC 17111 and DSM 40455^T^ were radically different in biological properties in terms of anticancer activities. The supernatant of CGMCC 17111 exhibited potent suppressive effects on HeLa (cervical cancer), ES-2 (ovarian cancer), A549 (lung cancer), and MC38 (colorectal cancer) cell lines, but not on the non-cancerous human gut epithelial NCM460 cells, demonstrating the high selectivity of CGMCC 17111 toxicity on malignant but not on non-cancerous cells; on the other hand, *S. albidoflavus* DSM 40455^T^ did not exhibit suppressive effects on any of these cancer cell lines (Fig. 2A and B; Fig. S1).

Flow cytometry analysis demonstrated that CGMCC 17111 significantly induced apoptosis in HeLa, A549, ES-2, and MC38 cells, whereas *S. albidoflavus* DSM 40455^T^ exhibited no pro-apoptotic effects on these cell lines (Fig. 2C and D). In addition to significantly increasing the sub-G1 population, which reflects apoptosis, CGMCC 17111 also caused S-phase accumulation in HeLa and A549 cells (Fig. 2E and F), suggesting DNA replication interference. These findings imply checkpoint activation at the G1/S transition. In contrast, *S. albidoflavus* DSM 40455^T^ merely induced reversible G1 arrest in MC38 cells and S-phase accumulation in NCM460 cells without triggering apoptosis.

### CGMCC 17111 inhibits colorectal cancer *in vivo*

**Fig 3:**
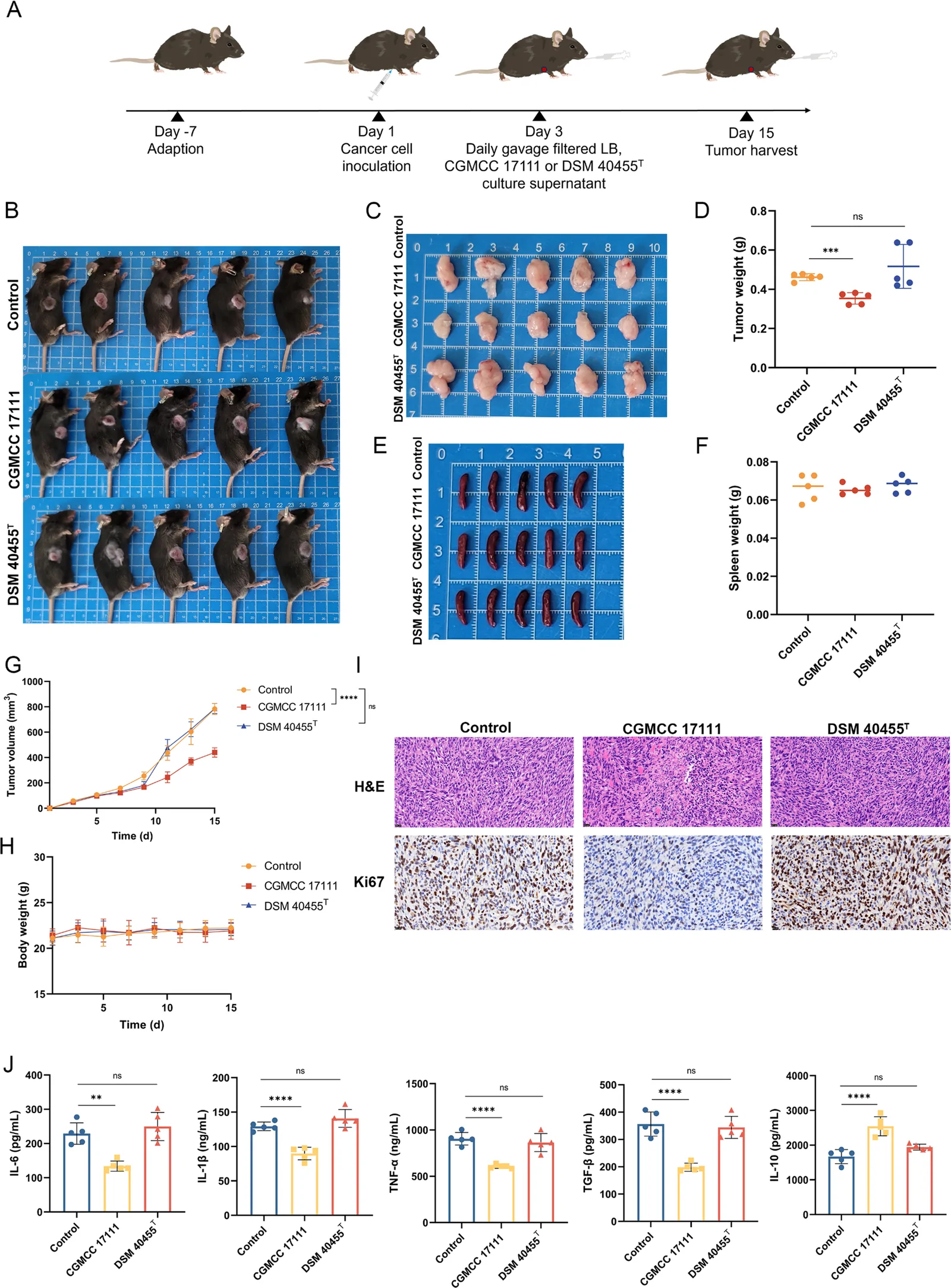
Tumor growth suppression *in vivo* by the culture of CGMCC 17111 (*n* = 5). (**A**) Experimental design of tumor inoculation and oral gavage in mice. (**B**) Syngeneic tumor models were established by transplanting MC38 cells into C57BL/6JNifdc mice. (**C**) Tumor growth at day 15 in the CGMCC 17111 culture supernatant-treated and DSM 40455^T^ culture supernatant-treated and control groups. (**D**) The removed tumors were weighed and statistically analyzed. The data represent the means ± SD. (**E**) Gross morphology of the spleen. (**F**) Statistical comparison of spleen weight among the animals of the three groups. The data represent the means ± SD. No significant differences were seen between CGMCC 17111 and DSM 40455^T^-treated animals and controls. The *P* values were 0.9505 and 0.8433, respectively. (**G**) Curves for tumor volumes during treatment with LB broth, CGMCC 17111 supernatant, and DSM 40455^T^ supernatant. (**H**) Curves for mouse weights during treatment with LB broth, CGMCC 17111 supernatant, or DSM 40455^T^ supernatant. (**I**) H&E staining and immunohistochemical staining (Ki-67) of tumor sections. Magnification, ×400. Scale bar, 20 µm. H&E, hematoxylin and eosin. ns, *P* > 0.05; *P < 0.05, **P ≤ 0.01, ***P ≤ 0.001, ****P < 0.0001 vs control by one-way ANOVA (**D, F, and J**) or by two-way ANOVA (**G and H**).

To evaluate the anticancer effect of CGMCC 17111 *in vivo*, we established a syngeneic tumor model by subcutaneously inoculating MC38 cells into C57BL/6JNifdc mice. The mice were divided into three groups and treated orally with sterile LB medium (control), CGMCC 17111 culture supernatant, or *S. albidoflavus* DSM 40455^T^ culture supernatant (Fig. 3A). Compared to control, CGMCC 17111 culture supernatant treatment remarkably suppressed tumor growth in volume and weight, while *S. albidoflavus* DSM 40455^T^ culture supernatant showed no antitumor effect (Fig. 3B through D and G). Importantly, no significant differences were found in spleen or body weight among mice of the groups (Fig. 3E, F, and H), indicating little or no harm of the anticancer compounds to the general health status of the mice. Histopathological analysis revealed evident tumor cell apoptosis and markedly reduced Ki-67 expression in the CGMCC 17111 culture supernatant-treated mice, whereas *S. albidoflavus* DSM 40455^T^ did not show such cancer-suppression effects (Fig. 3I). Furthermore, ELISA assays demonstrated that CGMCC 17111 culture supernatant treatment significantly decreased serum levels of pro-inflammatory cytokines (IL-6, IL-1β, TNF-α, and TGF-β) while elevating anti-inflammatory IL-10 (Fig. 3J). These results indicate that CGMCC 17111 exerts potent anticancer effects by inhibiting proliferation, promoting apoptosis, modulating immune responses, and inhibiting inflammation, without compromising host health.

### CGMCC 17111 alters the gut microbiome toward a healthier state

**Fig 4:**
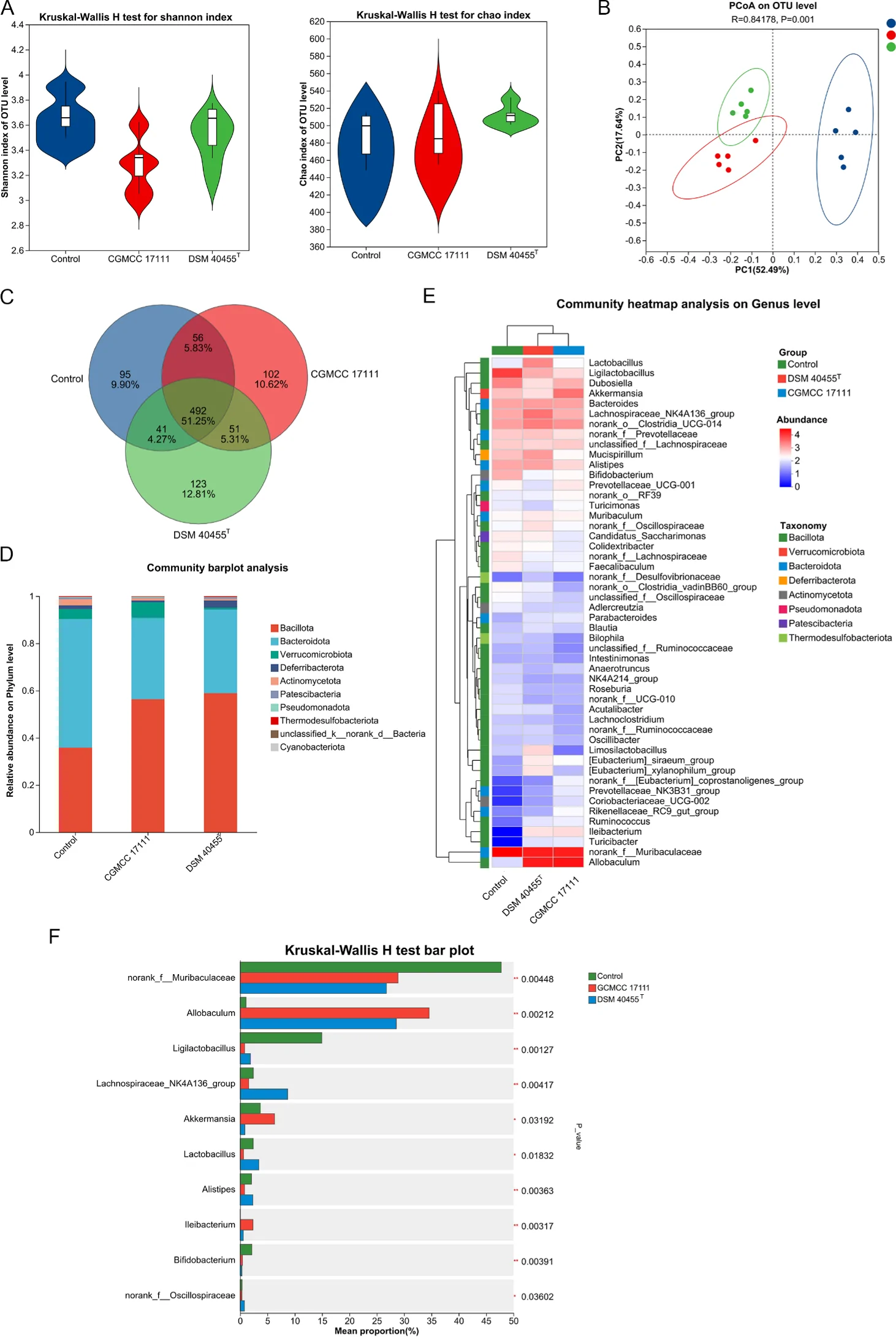
16S rDNA analysis unveiling gut microbiome composition of CGMCC 17111 compared with *S. albidoflavus* DSM 40455^T^ in C57BL/6JNifdc mice (*n* = 5). (**A**) The α-diversity of the fecal microbiome as illustrated by Shannon’s index and Chao index. (**B**) The β-diversity of the fecal microbiome demonstrated by a principal coordinates analysis (PCoA) plot. (**C**) Venn diagram of the three groups at the OTU level. (**D**) Relative abundances of abundant gut bacteria at phylum level. (**E**) Heatmap to visualize the relative abundances of the 30 most predominant bacterial genera. (**F**) Differential abundance analysis of gut bacterial genera among the three groups based on the Kruskal–Wallis *H* test. *P < 0.05, vs control by Student’s *t* test (**A**).

We conducted 16S rRNA gene sequencing and analysis on fecal specimens to assess the potential impact of CGMCC 17111 on the community structure of gut bacteria. CGMCC 17111 culture supernatant treatment led to a decrease in the Shannon index, while the Chao index remained unchanged (Fig. 4A), suggesting that CGMCC 17111 reshaped the gut microbiota by inhibiting certain pro-inflammatory or pathogenic bacteria while promoting the growth of relevant beneficial bacteria, thereby driving the microbial community toward a healthier state. The PCA analysis showed distinct clustering among the three groups (Fig. 4B). The Venn diagram showed 492 operational taxonomic units (OTUs) shared among the groups, 95 OTUs specific to the control group, 102 OTUs specific to the CGMCC 17111 culture supernatant treatment group, and 123 OTUs specific to the *S. albidoflavus* DSM 40455^T^ culture supernatant treatment group (Fig. 4C). At the phylum level, CGMCC 17111 culture supernatant-treated mice exhibited a remarkable increase in Verrucomicrobiota abundance compared to both control and *S. albidoflavus* DSM 40455^T^ groups, while in the meantime showing markedly decreased levels of Bacteroidota and Deferribacterota (Fig. 4D). At the genus level, mice of the CGMCC 17111 culture supernatant treatment group exhibited increased abundance of potentially beneficial taxa, including *Akkermansia* and *Allobaculum*, while, at the same time, showing a significant reduction in pro-inflammatory genera such as *Alistipes* (Fig. 4E and F), suggesting fine-tuning of the phylogenetic structure of gut bacteria by CGMCC 17111 toward amelioration of the composition and function of the gut microbiome. These results underscore the clinical application potential of CGMCC 17111 as a novel microbial therapeutic.

### CGMCC 17111 alters protein profiles of the cancer tissue to redress malignancy

**Fig 5:**
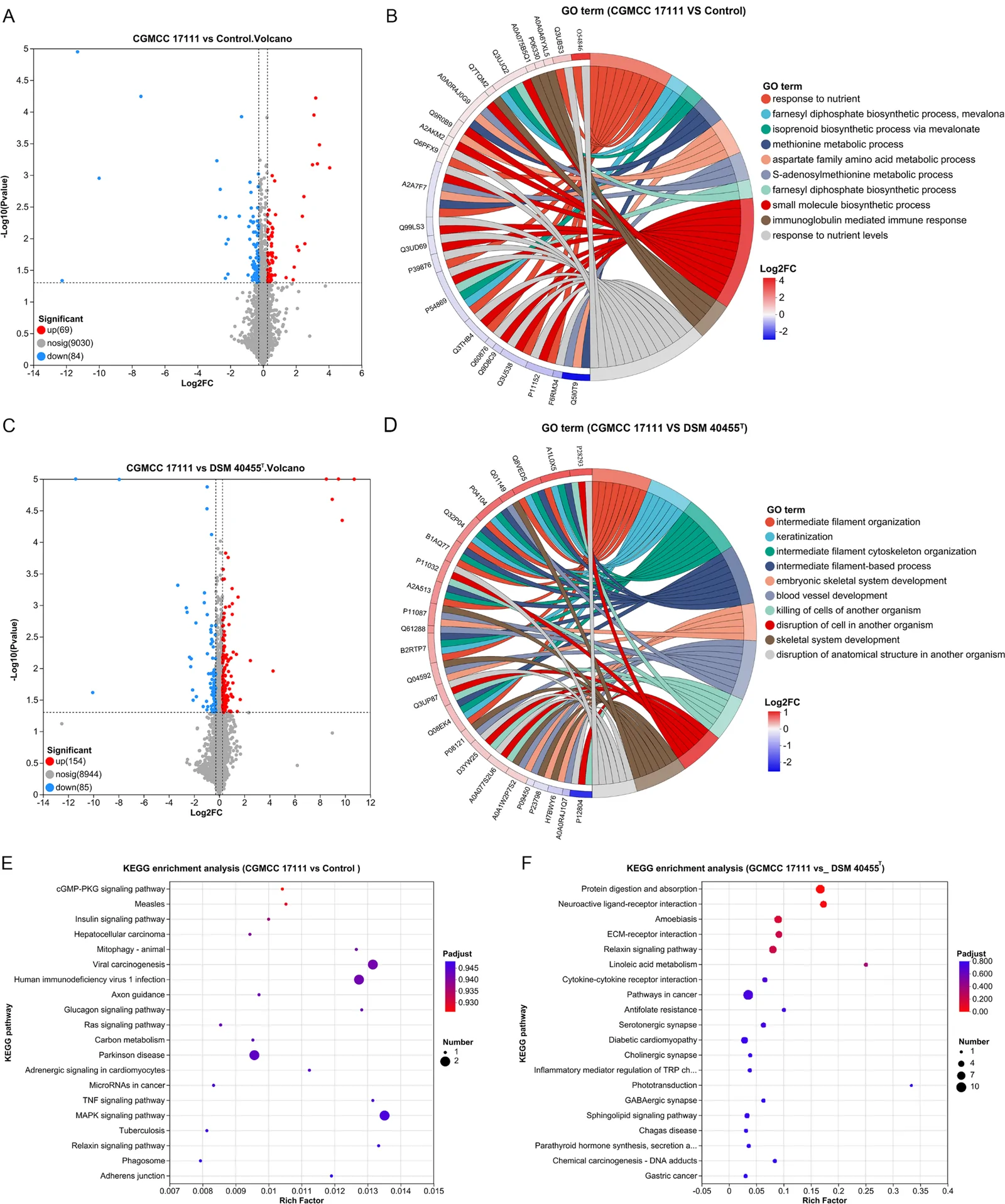
Proteomic analysis unveiling tumor protein expression alterations induced by CGMCC 17111 compared with *S. albidoflavus* DSM 40455^T^ in C57BL/6JNifdce mice (*n* = 5). (**A**) Volcano plot for differential proteins between CGMCC 17111 and control. (**B**) Volcano plot for differential proteins between CGMCC 17111 and DSM 40455^T^. (**C**) GO enrichment analysis of differentially expressed proteins between CGMCC 17111 and control. (**D**) GO enrichment analysis of differentially expressed proteins between CGMCC 17111 and DSM 40455^T^. (**E**) KEGG enrichment analysis of differentially expressed proteins of CGMCC 17111 vs control. (**F**) KEGG enrichment analysis of differentially expressed proteins of CGMCC 17111 vs DSM 40455^T^.

We employed the 4D-DIA technique to profile proteins within the cancer tissues in mice across the three groups. Compared with control, CGMCC 17111 culture supernatant-treated mice had a total of 153 differentially expressed proteins (DEPs), of which 69 were upregulated and 84 were downregulated (Fig. 5A). Compared with *S. albidoflavus* DSM 40455^T^, CGMCC 17111 culture supernatant-treated mice had a total of 239 DEPs, of which 154 were upregulated and 85 were downregulated (Fig. 5B). The GO enrichment string plot indicated that, compared to control, the CGMCC 17111 culture supernatant-treated mice were significantly enriched in methionine/SAM metabolism and mevalonate pathways (Fig. 5C). In addition, several biological processes associated with tissue remodeling and tumor-related biological responses, including intermediate filament assembly and angiogenesis-related pathways, were enriched in mice receiving CGMCC 17111 culture supernatant compared with DSM 40455^T^ (Fig. 5D) (12, 13). KEGG enrichment analysis further identified significant enrichment of pathways related to Ras signaling, MAPK signaling, and TNF signaling in the CGMCC 17111 culture supernatant group compared with the control group (Fig. 5E). Similarly, pathways associated with ECM–receptor interaction and chemical carcinogenesis–DNA adducts were significantly enriched in the CGMCC 17111 culture supernatant group compared with the DSM 40455T group (Fig. 5F). These enrichment results suggest that proteins associated with these biological pathways were differentially expressed following CGMCC 17111 treatment. However, enrichment analyses are descriptive in nature and should not be interpreted as direct evidence of pathway activation or causality. Further targeted studies will be required to validate the biological significance of these observations.

### Genomic and phylogenetic evidence for the distinctiveness of CGMCC 17111 from *S. albidoflavus*

**Fig 6:**
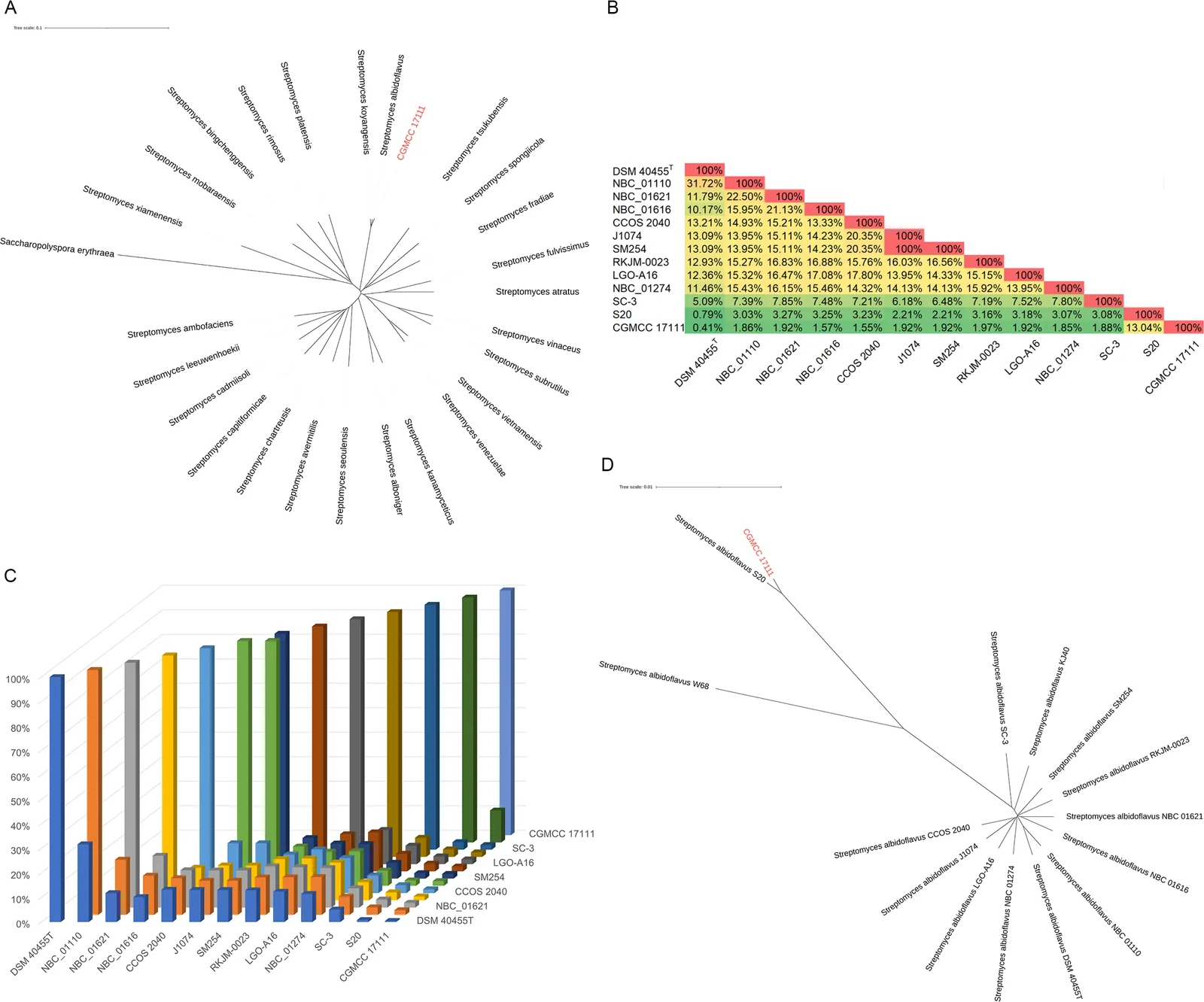
Genomic comparison among the *Streptomyces* strains. (**A**) Neighbor-joining tree for CGMCC 17111 and 26 sequenced *Streptomyces* type strains based on whole-genome sequences (all conserved regions among the compared genomes are concatenated and aligned for tree construction). *Saccharopolyspora erythraea* NRRL4338^T^ was used as outgroup. Bar, 0.01 substitutions per site. (**B**) Ratios of common genes with zero nucleotide degeneracy between pairs of strains compared. (**C**) Bar illustration of “B” for an intuitive view. (**D**) Phylogenetic relationships of CGMCC 17111 with *S. albidoflavus* strains based on conserved sequences. Bar, 0.01 substitutions per site.

Given the sharp contrast in anticancer activity between CGMCC 17111 and *S. albidoflavus* DSM 40455^T^, it is fundamental to determine the phylogenetic status of CGMCC 17111, especially its relationship with *S. albidoflavus*. The core issue is to determine whether CGMCC 17111 is an anticancer variant of *S. albidoflavus* or represents an independent species with a clear-cut genetic boundary with *S. albidoflavus*. We constructed a core genome phylogenetic tree based on shared genes from the type strains of 26 *Streptomyces* species (Fig. 6A), which was consistent with that of the 16S rDNA phylogenetic tree (see Fig. 1), further confirming that CGMCC 17111 is most closely related to *S. albidoflavus*. GenBank genome accession numbers are provided in Table S1.

We then focused on the sequenced strains of *S. albidoflavus* and found that most compared *S. albidoflavus* strains were genetically cohesive, forming a relatively tight cluster, except for strain S20 (Fig. 6B and C). Very interestingly, CGMCC 17111 was much more closely related to S20 than to the other *S. albidoflavus* strains compared, sharing 74.63% of the genes with S20; in contrast, CGMCC 17111 shares only 69.65% or an even lower percentage of its genes with the other *S. albidoflavus* strains, such as 66.74% with *S. albidoflavus* DSM 40455^T^. Among the compared strains of *S. albidoflavus*, the proportions of shared genes were all >90% except strain S20, which is much lower than 90% (85.65% between S20 and DSM 40455^T^) but still significantly higher than the 66.74% as between CGMCC 17111 and DSM 40455^T^ (Table S2).

Finally, we needed to determine whether the genetic boundary may exist between CGMCC 17111 and *S. albidoflavus* by conducting systematic sequence comparisons of individual genes shared between pairs of CGMCC 17111 and the *S. albidoflavus* strains. Previously, genetic boundary, defined as low ratios (10% or lower) of total homologous genes with zero sequence degeneracy between bacteria in comparison, has been proposed to delineate closely related bacteria into natural species (9–11, 14). Consistent with the low proportions of total shared genes between CGMCC 17111 and the *S. albidoflavus* strains, ratios of total homologous genes with zero sequence degeneracy between CGMCC 17111 and the *S. albidoflavus* strains were sufficiently low as to reflect the existence of genetic boundaries between CGMCC 17111 and the *S. albidoflavus* strains (Fig. 6B and C) and separate CGMCC 17111 from *S. albidoflavus* as representing a novel natural species.

Collectively, the phylogenetic analyses based on core genes delineated CGMCC 17111 and the *S. albidoflavus* strains into three distinct clades: the *S. albidoflavus* DSM 40455^T^ clade (11 strains analyzed in this study), a clade represented by CGMCC 17111, and a clade represented by S20 (Fig. 6D), with CGMCC 17111 representing a novel natural species and S20 probably representing another natural species.

### Functional annotation of the CGMCC 17111 genome

**Fig 7:**
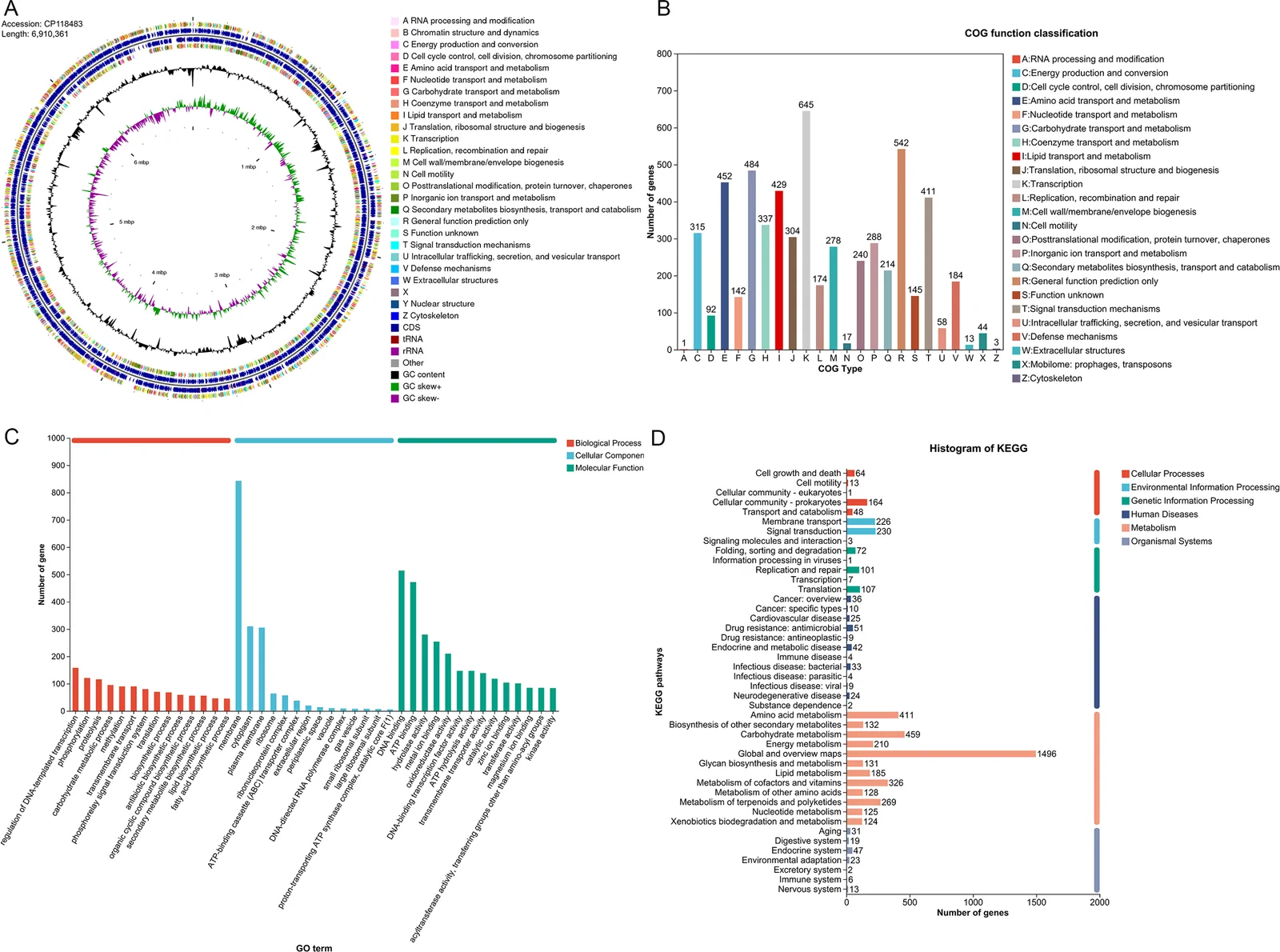
Whole-genome information for CGMCC 17111. (**A**) Circular diagram of the genome of CGMCC 17111 using CGView comparison tool. From outside to inside: circle 1, COG functional categories for forward strand coding sequences, and the different colors represent different COG categories; circle 2, forward strand sequence features; circle 3, reverse strand sequence features; circle 4, COG functional categories for reverse strand coding sequence; circle 5, GC content; and circle 6, GC skew. (**B**) COG functional gene classification of CGMCC 17111. (**C**) GO annotation. (**D**) KEGG clustering annotation.

To elucidate functional differences between CGMCC 17111 (representing a novel species) and its closest known relative (of a distinct species), we conducted whole-genome comparisons between CGMCC 17111 and *S. albidoflavus* DSM 40455^T^. The focus of the question is to understand their distinct genetic and biological traits that might have promoted their phylogenetic as well as phenotypic divergence, with CGMCC 17111 possessing potent anticancer activities but DSM 40455^T^ lacking appreciable impact on cancer. The CGMCC 17111 genome is a single closed circle, 6,910,361 bp in size, with a GC content of 73.03%, 7,556 coding sequences, 66 tRNA genes, and 21 rRNA genes (Fig. 7A). The high GC content (69%–78%) serves as a key taxonomic characteristic of the genus *Streptomyces* and so is used as a valuable molecular marker for this genus. According to the COG function classification (Fig. 7B), transcription and lipid or carbohydrate metabolism were among the most highly encoded functions. In the GO classification (Fig. 7C), molecular functions had the highest number of genes (3,513), followed by biological processes (1,948) and cellular components (1,630). Among the six KEGG pathways (Fig. 7D), metabolism contained the highest number of genes (3,996 genes), followed by environmental information processing (459 genes). Genetic and functional analysis of CGMCC 17111 identified a secondary metabolite biosynthetic gene cluster (Cluster 3, T1PKS) showing 83% similarity to the angucycline-class antibiotic SGR PTMs (Table S3) (15). Because several angucycline-related compounds have previously been reported to possess antitumor activity, the presence of this biosynthetic gene cluster suggests that CGMCC 17111 may have the potential to produce bioactive secondary metabolites. However, the specific metabolites produced by this cluster and their contribution to the observed antitumor effects remain unknown and require further experimental validation.

### Polyphasic characterization of CGMCC 17111

To reflect the existence of the genetic boundary between CGMCC 17111 and *S. albidoflavus* DSM 40455^T^ by systematic comparisons of their biological properties, we carried out polyphasic taxonomical studies on the two strains. CGMCC 17111 grew well on LB, R_2_A (16), Gause’s synthetic no. 1, and Streptomyces Project (ISP) 1–5 agar media. On the R2A agar plates, the colonies formed yellow substrate hyphae and abundant white aerial, with no detectable pigment production (Fig. S2A and B). The aerial mycelium of CGMCC 17111 was divided into straight or curved chains of spores (Fig. S2C). CGMCC 17111 formed ovoid to cylindrical spores, smooth, and 1.0–1.3 μm in diameter. In contrast, the aerial mycelia of *S. albidoflavus* DSM 40455^T^ proliferated well on most of the culture media, with the substrate mycelia and aerial mycelia being in white color without soluble pigments (Fig. S2D and E).

Both strains had a growth temperature range of 25–40°C, with 37°C being optimal for CGMCC 17111 but 28°C for *S. albidoflavus* DSM 40455^T^, indicating the former adapted to warm-blooded hosts such as humans and the latter an environmental microbe. NaCl tolerance was up to 8% (wt/vol) for both strains. They also both presented positive results for urea hydrolysis and nitrate reduction and hydrolyzed cellulose. They both tested negative for H_2_S production but showed differences in carbon metabolism (D-trehalose, α-D-lactose, etc.; Table S4), enzyme profiles (α-galactosidase, β-galactosidase), and functional traits (starch degradation, milk coagulation).

### Chemotaxonomic characterization

CGMCC 17111 and *S. albidoflavus* DSM 40455^T^ both contained LL-diaminopimelic acid, predominantly found in their cell walls. Ribose, xylose, and arabinose were detected in whole-­cell hydrolysates of CGMCC 17111, while *S. albidoflavus* DSM 40455^T^ only contained ribose. The polar lipid profile of CGMCC 17111 consisted of phosphatidylethanolamine (PE), phosphatidylmonomethylethanolamine (PME), unknown glycolipids (GL), unknown aminolipid (AL), and unidentified polar lipids (L), whereas *S. albidoflavus* DSM 40455^T^ contained PE but not PME. CGMCC 17111 was comprised of iso-C16:0 (27.51%) and anteiso-C15:0 (21.59%), whereas *S. albidoflavus* DSM 40455^T^ showed anteiso-C15:0 (28.77%), C16:0 (14.25%), and iso-C15:0 (13.73%) (Table S5). The major menaquinones of CGMCC 17111 consisted of MK-9 (H6 and H8).

## DISCUSSION

The present study demonstrates that culture supernatant derived from the human gut isolate *Streptomyces* sp. CGMCC 17111 exhibits antitumor activity both *in vitro* and *in vivo*. Treatment with CGMCC 17111 culture supernatant inhibited the growth of multiple cancer cell lines, promoted apoptosis, altered cell cycle progression, reduced tumor growth in the MC38 mouse model, and was associated with changes in inflammatory cytokines, gut microbial composition, and tumor-associated protein expression profiles. Collectively, these findings suggest that bioactive factors secreted by CGMCC 17111 may contribute to tumor suppression and modulation of the host microenvironment.

Our findings further support the concept that the human gut microbiota may contain bacterial strains with previously unrecognized antitumor potential, consistent with our previous observations (8, 17). However, the prevalence, ecological significance, and functional contribution of such bacteria within the human gut ecosystem remain largely unknown. Numerous environmental and host-associated factors, including antimicrobial exposure, dietary patterns, smoking, psychological stress, and sleep disruption, have been reported to influence gut microbial composition and function (6). Whether these factors also affect the abundance or activity of putative antitumor bacteria warrants further investigation.

Genome-based analyses indicated that CGMCC 17111 is genetically distinct from its closest currently described relative, *S. albidoflavus* DSM 40455^T^. Although the two strains share high 16S rRNA gene sequence similarity, whole-genome comparisons revealed clear genetic divergence based on ANI, dDDH, and core-genome analyses. Therefore, CGMCC 17111 appears to represent a genetically distinct *Streptomyces* lineage. Nevertheless, additional taxonomic and evolutionary studies will be necessary to further clarify its phylogenetic status and relationship to currently recognized *Streptomyces* species.

The genomic and phenotypic differences between CGMCC 17111 and DSM 40455^T^ are particularly noteworthy. In addition to differences in chemotaxonomic characteristics, only CGMCC 17111 exhibited reproducible antitumor activity in both cell culture and animal experiments. Genome mining identified several biosynthetic gene clusters with the potential to produce bioactive secondary metabolites. However, the active molecule(s) responsible for the observed antitumor effects remain unknown. Although a T1PKS-containing biosynthetic gene cluster showed similarity to known angucycline-associated pathways, the relationship between this cluster and the observed biological phenotype remains speculative and requires experimental validation through metabolite characterization and functional genetic studies.

Alterations in the gut microbiota were observed following administration of CGMCC 17111 culture supernatant, including changes in several bacterial taxa previously associated with intestinal homeostasis and inflammatory responses. These findings raise the possibility that modulation of the gut microbial ecosystem may contribute to the observed antitumor phenotype. However, the current study cannot distinguish whether tumor suppression resulted primarily from direct actions of CGMCC 17111-derived metabolites on host tissues, indirect effects mediated through microbiota remodeling, or a combination of both mechanisms. Future studies involving microbiota transplantation and mechanistic validation will be required to address these questions.

Proteomic analyses identified differential expression of proteins associated with methionine/SAM metabolism, mevalonate metabolism, Ras signaling, MAPK signaling, TNF signaling, and extracellular matrix-related pathways. Methionine metabolism and its derivative S-adenosylmethionine (SAM) are central regulators of cellular methylation reactions and epigenetic modifications, which have been implicated in tumor initiation and progression. Likewise, the mevalonate pathway contributes to cholesterol biosynthesis, protein prenylation, and regulation of cell proliferation and survival. Alterations in proteins associated with these pathways may, therefore, reflect changes in tumor metabolic and regulatory processes following CGMCC 17111 culture supernatant treatment. However, enrichment analyses are descriptive in nature and should be interpreted as hypothesis-generating observations rather than direct evidence of pathway activation or causality. Additional targeted studies will be required to determine the biological significance of these findings and their relationship to the antitumor effects of CGMCC 17111.

Several limitations of the present study should be acknowledged. First, the biological activity evaluated in this work was derived from sterile culture supernatant rather than live bacterial administration. Consequently, the contribution of intestinal colonization and host–microbiota interactions remains unclear. Second, the active antitumor factors present in the culture supernatant have not yet been identified. Third, antitumor efficacy was evaluated in a single MC38 tumor model, and broader validation in additional tumor models will be required. Finally, although CGMCC 17111 culture supernatant demonstrated selective inhibitory effects against multiple cancer cell lines while showing minimal effects on the non-cancerous intestinal epithelial cell line NCM460, comprehensive toxicological evaluation was not performed. Therefore, further studies incorporating metabolite identification, mechanistic validation, histopathological examination, and systematic safety assessment will be necessary before therapeutic applications can be considered.

In conclusion, CGMCC 17111 represents a genetically distinct gut-derived *Streptomyces* strain whose culture supernatant exhibits selective antitumor activity *in vitro* and suppresses tumor growth *in vivo*. These findings expand our understanding of the functional diversity of gut-associated actinomycetes and provide a foundation for future investigations into the bioactive metabolites and molecular mechanisms underlying their antitumor properties.

## MATERIALS AND METHODS

### Bacterial strains

Human fecal samples were collected from healthy donors and cultured under appropriate conditions. Bacterial isolates were individually cultured, and sterile-filtered culture supernatants were prepared. Anticancer activity was initially screened by evaluating the effects of culture supernatants on cancer cell viability. Strains showing significant growth-inhibitory activity were subjected to secondary validation. Among the candidate strains, CGMCC 17111 consistently exhibited the strongest and broadest antitumor activity and was, therefore, selected for further characterization. *S. albidoflavus* DSM 40455^T^ was acquired from the Shanghai Bioresource Collection Center (SHBCC) (18). Both strains were cultured under identical conditions.

### Whole-genome sequencing

Genomic DNA from CGMCC 17111 was isolated with the Wizard Genomic DNA Purification Kit (Promega), following the supplier’s guidelines (19). *De novo* assembly of high-quality reads into scaffolds was performed using SOAPdenovo2 (20). Glimmer v3.02 was used for predicting coding sequences (CDs) (21). tRNA-scan-SE v1.3.1 was used for tRNA prediction. Barrnap v0.4.2 was used for rRNA prediction. The predicted CDSs were annotated by NCBI nr, Swiss-Prot, Pfam, COG, GO, and KEGG databases using sequence alignment tools BLASTP, Diamond, and HMMER (22). Each set of query proteins was aligned with the databases, and annotations of the best-matched subjects (*e*-value < 10^−5^) were obtained for gene annotation.

### Phylogenetic analysis and molecular studies

The 16S rRNA gene sequence for CGMCC 17111 was extracted from whole-genome sequence and identified on the EzBioCloud server (https://www.ezbiocloud.net/). 16S rRNA gene sequence similarities between CGMCC 17111 and type strains were calculated using the EzBioCloud server. Multiple sequence alignment was conducted via the BioEdit v7.2 program. The neighbor-joining method was used to reconstruct phylogenetic trees using the MEGA v11.0 software. The robustness of the phylogenetic trees was calculated by bootstrap analyses based on 1,000 random replications of the sequences. The final tree was generated using the iTOL v7 (https://itol.embl.de/) tool. Average nucleotide identity was calculated using the EZ BioCloud ANI calculator. Digital DNA-DNA hybridizations were performed using the web server Genome-to-Genome Distance Calculator (GGDC) v 2.1, with BLAST as a local alignment tool (18).

### Cell lines and cytotoxic activity assays

The human cancer cell lines HeLa (cervical carcinoma), A549 (non-small cell lung cancer), ES-2 (ovarian clear cell carcinoma), MC38 (colorectal cancer), and the non-tumorigenic NCM460 line were maintained as described previously (18). HeLa, A549, and MC38 cells were grown in DMEM or RPMI 1640 supplemented with 10% fetal bovine serum (FBS), while ES-2 cells were cultured in McCoy’s 5A medium with 10% FBS. All the cell lines were cultured at 37°C in a humidified atmosphere with 5% CO_2_. Single colonies of CGMCC 17111 or *S. albidoflavus* DSM 40455^T^ were picked and inoculated in LB broth and incubated at 37°C and 150 rpm in a rotary shaker incubator. After 9 days, the bacterial culture was centrifuged at 12,000 rpm for 2 min, and the supernatant was filtered through a 0.22 filter membrane and used for cancer cell treatment. Cell viability assays were performed using the Cell Counting Kit-8 (CCK-8; APExBIO, America, K1018). After centrifugation, the cells were plated in 96-well plates at 10^4^ cells per well, cultured overnight, and treated with different dilutions of the filtered bacterial supernatant. After 24 h, the morphological changes in the cells were inspected by phase-contrast inverted microscopy (Primo Star; Carl Zeiss), and 10 μL of CCK-8 solution was added to each well. The plate was detected using a microplate reader at 450 nm. Cell survival rate (%) = [(*A* (experimental group) − *A* (blank)]/[(*A* (control group) − *A* (blank)] × 100%.

### Apoptosis assay

Cells exposed to bacterial supernatants for 24 h were collected, counted, and stained with annexin V-FITC and propidium iodide (PI) using a commercial apoptosis detection kit. After incubation in the dark, samples were analyzed by flow cytometry (NovoCyte, Agilent) within 1 h (18). Q3-1 indicates necrotic cells (annexin V−/PI+), Q3-2 indicates late apoptotic cells (annexin V+/PI+), Q3-3 indicates viable cells (annexin V−/PI−), and Q3-4 indicates early apoptotic cells (annexin V+/PI−). The total apoptotic rate was calculated as Q3-2 + Q3-4 from three independent experiments.

### Cell cycle analysis

Treated cells were washed with PBS, fixed, and stained with PI solution. Following incubation at 37°C for 30 min in the dark, DNA content was analyzed by flow cytometry to determine cell cycle distribution (18).

### *In vivo* study

Male C57BL/6JNifdce mice at 6 weeks of age were purchased from Beijing Vital River Laboratory Animal Technology Co., Ltd., and housed in a specific pathogen-free facility. MC38 cells (6.0 × 10⁵ in 0.1 mL PBS) were injected subcutaneously. Once tumors were palpable, mice were randomized into groups (*n* = 5) and administered daily oral gavage of 0.5 mL sterile supernatant from CGMCC 17111, *S. albidoflavus* DSM 40455^T^, or LB broth until the endpoint. Tumor dimensions were measured every other day, and volume was calculated as *V* = *L* × *W*^2^ × 1/2, where *V* is the volume, *L* is the length, and *W* is the width. At the end of the experiment, the mice were weighed and sacrificed, and the tumors were dissected, weighed, and stored in 4% paraformaldehyde solution for hematoxylin-eosin staining (8).

### Histopathological examination

Tumor tissue from mice was separated, and a section of it was rapidly frozen in 4% paraformaldehyde solution for histologic examination. Hematoxylin and eosin (H&E) staining was conducted by Servicebio Biotechnology Co., Ltd. (Wuhan, China) (8). The expression of the proliferation marker Ki-67 in tumor tissues was assessed by immunohistochemistry (IHC). The levels of interleukin-1 beta (IL-1β; CAS:MM-0040M1, mmbio, China), interleukin-10 (IL-10; CAS:MM-0176M1, mmbio, China), interleukin-6 (IL-6; CAS:MM-0163M1, mmbio, China), tumor necrosis factor-alpha (TNF-α; CAS:MM-0132M1, mmbio, China), and transforming growth factor-beta (TGF-β; CAS:MM-0689M1, mmbio, China) were quantified using commercial ELISA kits according to manufacturer instructions.

### 16S rRNA sequencing and data analysis

Fresh mouse fecal samples were collected, flash-frozen in liquid nitrogen, and stored at −80°C. Fecal DNA was extracted with the FastPure Stool DNA Isolation Kit (MJYH, Shanghai, China). The V3–V4 region of the 16S rRNA gene was amplified with primers. The 16S rDNA sequences were clustered into operational taxonomic units (OTUs) using UPARSE 7.1 with 97% sequence similarity (23). The taxonomy of each OTU sequence was analyzed by RDP Classifier version 2.2 against the 16S rRNA gene database using a confidence threshold of 0.7 (24). The metagenomic function was predicted by PICRUSt2 (Phylogenetic Investigation of Communities by Reconstruction of Unobserved States) based on the OTU sequences (25).

### 4D-DIA proteomic analysis

Proteomic analysis of tumor tissues from different groups was conducted by Majorbio Bio-Pharm Technology Co., Ltd., of China. Tumor tissue proteins were extracted, digested, and analyzed by liquid chromatography-tandem mass spectrometry (26). Data acquisition used DIA-PASEF, and spectra were processed with Spectronaut 14.0. Differential expression analysis was conducted on the Majorbio Cloud platform (https://cloud.majorbio.com) (27). *P*-values and fold change (FC) for the proteins between the two groups were calculated using the R package “*t*-test.” The thresholds of fold change (>1.2 or <0.83) and *P*-value <0.05 were used to identify differentially expressed proteins (26).

### Core genome-based phylogenetic analysis

We concatenated the sequences of genes common to the bacterial strains analyzed and conducted comparisons using the Basic Local Alignment Search Tool (BLAST) with the parameters set at >70% DNA identity and >70% gene length to categorize genes into common genes (18, 28). Individual orthologous sequences were aligned by the MAFFT program (29). The phylogenetic trees of the genomes were structured using the neighbor-joining method in MEGA 11.0 (30) by 1,000 bootstrap replicates. The evolutionary distances were computed using the *p*-distance method (31).

### Growth conditions and phenotypic characterization

The colonial morphology of strain CGMCC 17111 and *S. albidoflavus* DSM 40455^T^ was examined after incubation at 37°C for 7 days on various agar media, including ISP 1–ISP 5, R_2_A, LB, and Gause’s synthetic agar no. 1. Hyphal and spore chain structures were documented using the coverslip technique (32). Morphological observations were carried out with both light microscopy (Primo Star, Carl Zeiss, Germany) and scanning electron microscopy (Quanta 450, FEI, USA) following 7 days of growth on R_2_A. The temperature range for growth was tested at 4°C, 9°C, 19°C, 28°C, 37°C, 40°C, and 50°C. Tolerance to pH was assessed at intervals from pH 3 to 12 using citrate buffer (pH 3.0–5.0), phosphate buffer (pH 6.0–8.0), carbonate-bicarbonate buffer (pH 9.0–10.0), and disodium phosphate–sodium hydroxide buffer (pH 11.0–12.0). Salt tolerance was evaluated on R2A medium supplemented with NaCl at concentrations ranging from 0% to 10.0% (wt/vol). Starch degradation was tested on R_2_A containing 0.2% soluble starch. Biochemical traits including H₂S production, urea hydrolysis, cellulose hydrolysis, and milk coagulation were assessed according to Ruan et al. (33). Additional enzymatic profiles were determined using API ZYM kits (CAS: 25,200, bioMérieux, France) under optimal conditions. Oxidation of organic substrates was analyzed with the GEN III MicroPlate system (CAS: 1030, Biolog, USA) following incubation at 37°C (18).

### Chemotaxonomic characterization

Biomass for chemical analysis was obtained from cultures grown in trypticase soy broth at 37°C for 14 days under shaking conditions. Cells were harvested by centrifugation and washed with distilled water. Diaminopimelic acid (DAP) (34) and whole-cell sugars were analyzed by thin-layer chromatography (TLC) (35). The washed cells (100 mg) were saponified, methylated, and extracted. Fatty acid methyl esters were prepared from 100 mg of harvested cells via saponification, methylation, and extraction according to the standard MIDI protocol (Sherlock Microbial Identification System, version 6.0B) (18). Analysis was performed using gas chromatography (Agilent Technologies 6850) with reference to the TSBA6.0 database. Isoprenoid quinones were identified by reversed-phase high-performance liquid chromatography (HPLC) (18). Polar lipids were extracted from freeze-dried cells (100 mg) and separated via two-dimensional TLC on 10 × 10 cm plates (Merck). Specific detection of aminolipids and phospholipids was achieved using ninhydrin and molybdenum blue reagents, respectively, after heating at 120°C for 5–10 min (36).

### Statistical analysis

All statistical analyses were performed using GraphPad Prism 9.5, and Photoshop was used for illustrations. The data from at least three independent experiments were presented as mean ± standard deviations (mean ± SD). The statistics were performed using the student’s *t*-test or the Mann–Whitney *U* test for comparison between two groups, and the ANOVA test was used to compare statistical significance among multiple groups. Differences were considered significant at *P* < 0.05.

## Data Availability

The sequenced genome of CGMCC 17111 was deposited in NCBI GenBank Database under BioProject PRJNA891903 with the accession number CP118483 (https://www.ncbi.nlm.nih.gov/nuccore/CP118483.1/). The raw sequencing reads were deposited into the NCBI Sequence Read Archive (SRA) database (accession numbers: SRR34438204–34438220; https://www.ncbi.nlm.nih.gov/sra). The proteomics data are available in the ProteomeXchange repository under accession number PXD066301 (https://proteomecentral.proteomexchange.org/ui?pxid=PXD066301).

## SUPPLEMENTAL MATERIAL

Figure S1 msystems.00528-26-s0001.docx Morphology of different cancer cell lines after treatment with the culture supernatant of CGMCC 17111 and *S. albidoflavus* DSM 40455^T^.

Figure S2 msystems.00528-26-s0002.docx Comparisons of colony morphology on Gause's synthetic agar plate of CGMCC 17111 and *S. albidoflavus* DSM 40455^T^.

Table S1 msystems.00528-26-s0003.docx *Streptomyces* strains and GenBank genome accession numbers.

Table S2 msystems.00528-26-s0004.xlsx Numbers of genes in common between CGMCC 17111 and *S. albidoflavus* strains.

Table S3 msystems.00528-26-s0005.xlsx Secondary metabolite biosynthesis gene clusters of strain CGMCC 17111 predicted by antiSMASH.

Table S4 msystems.00528-26-s0006.docx Phenotypic properties of CGMCC 17111 and *S. albidoflavus* DSM 40455^T^.

Table S5 msystems.00528-26-s0007.docx Fatty acid composition of strain CGMCC 17111 and *S. albidoflavus* DSM 40455^T^.

ASM does not own the copyrights to Supplemental Material that may be linked to, or accessed through, an article. The authors have granted ASM a non-exclusive, world-wide license to publish the Supplemental Material files. Please contact the corresponding author directly for reuse.
