# Supplementary material for: A novel human Streptomyces strain redresses malignancy by inducing apoptosis and regaining gut homeostasis
Source: mSystems. 2026 Sep 23;11(10):e00528-26. doi: 10.1128/msystems.00528-26 (PMC13712931; doi:10.1128/msystems.00528-26)
Supplement: Supplementary file 1 [file msystems.00528-26-s0001.docx]

Figure S1. Morphology of different cancer cell lines after treatment with the culture supernatant of CGMCC 17111 and *S. albidoflavus* DSM 40455^T^.
