# Supplementary material for: A novel human Streptomyces strain redresses malignancy by inducing apoptosis and regaining gut homeostasis
Source: mSystems. 2026 Sep 23;11(10):e00528-26. doi: 10.1128/msystems.00528-26 (PMC13712931; doi:10.1128/msystems.00528-26)
Supplement: Supplementary file 2 [file msystems.00528-26-s0002.docx]

Figure S2. Comparisons of colony morphology on Gause’s Synthetic agar plate of CGMCC 17111 and *S. albidoflavus* DSM 40455^T^.

A: Morphological photograph of strain CGMCC 17111 on the front of the plate; B: Morphological photograph of strain CGMCC 17111 on the back of the plate; C: Scanning electron micrograph of strain CGMCC 17111. D: Morphological photograph of strain DSM 40455^T^ on the front of the plate; E: Morphological photograph of strain DSM 40455^T^ on the back of the plate.
