# Supplementary material for: A novel human Streptomyces strain redresses malignancy by inducing apoptosis and regaining gut homeostasis
Source: mSystems. 2026 Sep 23;11(10):e00528-26. doi: 10.1128/msystems.00528-26 (PMC13712931; doi:10.1128/msystems.00528-26)
Supplement: Supplementary file 3 [file msystems.00528-26-s0003.docx]

**Table S1. *Streptomyces* strains and GenBank genome accession numbers.**

| **Species** | **Type strain** | **Accession number** |
| --- | --- | --- |
| *Streptomyces albidoflavus* | DSM 40455 | PKLO01000004 |
| *Streptomyces alboniger* | NRRL B-1832 | CP023695 |
| *Streptomyces ambofaciens* | ATCC 23877 | CP012382 |
| *Streptomyces atratus* | NRRL B-16927 | CP027306 |
| *Streptomyces avermitilis* | MA-4680 | NC_003155 |
| *Streptomyces bingchenggensis* | BCW-1 | NC_016582 |
| *Streptomyces cadmiisoli* | ZFG47 | CP030073 |
| *Streptomyces capitiformicae* | 1H-SSA4 | CP022161 |
| *Streptomyces chartreusis* | NBRC 12753 | CP023689 |
| *Streptomyces fradiae* | DSM 40063 | CP023696 |
| *Streptomyces fulvissimus* | DSM 40593 | NC_021177 |
| *Streptomyces kanamyceticus* | NBRC 13414 | CP023699 |
| *Streptomyces koyangensis* | VK-A60 | CP031742 |
| *Streptomyces leeuwenhoekii* | C34 | LN831790 |
| *Streptomyces mobaraensis* | NBRC 13819 | CP072827 |
| *Streptomyces platensis* | JCM 4662 | CP023691 |
| *Streptomyces rimosus* | ATCC 10970 | CP023688 |
| *Streptomyces seoulensis* | NRRL B-24310 | CP032229 |
| *Streptomyces spongiicola* | HNM0071 | CP029254 |
| *Streptomyces subrutilus* | ATCC 27467 | CP023701 |
| *Streptomyces tsukubensis* | NRRL 18488 | CP020700 |
| *Streptomyces venezuelae* | ATCC 10712 | NC_018750 |
| *Streptomyces vietnamensis* | GIM4.0001 | CP010407 |
| *Streptomyces vinaceus* | NBRC 13414 | CP023692 |
| *Streptomyces xiamenensis* | MCCC 1A01550 | CP009922 |
| *Saccharopolyspora erythraea* | NRRL 2338 | AM420293 |
| *Streptomyces albidoflavus* | CCOS 2040 | OX371412.1 |
| *Streptomyces albidoflavus* | J1074 | CP004370.1 |
| *Streptomyces albidoflavus* | KJ40 | CP016824.1 |
| *Streptomyces albidoflavus* | LGO A16 | CP079112.1 |
| *Streptomyces albidoflavus* | NBC_01110 | CP108647.1 |
| *Streptomyces albidoflavus* | NBC_01274 | CP108447.1 |
| *Streptomyces albidoflavus* | NBC_01616 | CP109299.1 |
| *Streptomyces albidoflavus* | NBC_01621 | CP109294.1 |
| *Streptomyces albidoflavus* | RKJM-0023 | CP133227.1 |
| *Streptomyces albidoflavus* | S20 | CP170353.1 |
| *Streptomyces albidoflavus* | SC-3 | CP152102.1 |
| *Streptomyces albidoflavus* | SM254 | CP014485.1 |
| *Streptomyces albidoflavus* | W68 | CP064783.1 |
