# Supplementary material for: A novel human Streptomyces strain redresses malignancy by inducing apoptosis and regaining gut homeostasis
Source: mSystems. 2026 Sep 23;11(10):e00528-26. doi: 10.1128/msystems.00528-26 (PMC13712931; doi:10.1128/msystems.00528-26)
Supplement: Supplementary file 4 [file msystems.00528-26-s0006.docx]

**Table S4. Phenotypic properties of CGMCC 17111 and *S. albidoflavus*** **DSM 40455^T^**

| Characteristics | **CGMCC 17111** | **DSM 40455^T^** |
| --- | --- | --- |
| **Genotypic:** |  |  |
| Genome Size (Mb) | 6910 | 6969 |
| GC Content (%) | 73.03 | 74.77 |
| **Phenotypic:** |  |  |
| Colony Morphology (on Gause’s synthetic No. 1 media) | yellow substrate hyphae and abundant white aerial hyphae | white substrate hyphae and abundant white aerial hyphae |
| Temperature (°C) | 25-40 | 25-40 |
| pH | 5-12 | 5-12 |
| NaCl (%, w/v) | 0-8 | 0-8 |
| **Available media:** |  |  |
| LB | + | + |
| R_2_A | + | + |
| Gauze’s agar | + | + |
| ISP1 | + | + |
| ISP2 | + | + |
| ISP3 | + | + |
| ISP4 | + | + |
| ISP5 | + | + |
| Decomposition of Starch | - | + |
| Urea hydrolysis | + | + |
| Produces H_2_S | - | - |
| Nitrate reduction | + | + |
| Hydrolyzes cellulose | + | + |
| Coagulation of milk | + | - |
| **Assimilation of (Biolog GEN III):** |  |  |
| Dextrin | + | + |
| D-Maltose | + | + |
| D-Trehalose | - | + |
| D-Cellobiose | + | + |
| Gentiobiose | + | + |
| Sucrose | - | - |
| D-Turanose | - | - |
| Stachyose | - | - |
| D-Raffinose | - | - |
| α-D-Lactose | + | - |
| D-Melibiose | - | + |
| β-Methyl-D-Glucoside | + | - |
| D-Salicin | + | - |
| N-Acetyl-D-Glucosamine | + | + |
| N-Acetyl-β-DMannosamine | - | - |
| N-Acetyl-D-Galactosamine | - | + |
| N-AcetylNeuraminic Acid | + | - |
| α-D-Glucose | + | + |
| D-Mannose | + | + |
| D-Fructose | + | + |
| D-Galactose | + | + |
| 3-Methyl Glucose | - | - |
| D-Fucose | - | - |
| L-Fucose | - | + |
| L-Rhamnose | - | - |
| Inosine | + | + |
| 1% Sodium Lactate | + | + |
| Fusidic Acid | - | + |
| D-Serine | - | + |
| D-Sorbitol | - | - |
| D-Mannitol | + | + |
| D-Arabitol | + | + |
| myo-Inositol | - | + |
| Glycerol | + | + |
| D-Glucose-6-PO_4_ | - | + |
| D-Fructose-6-PO_4_ | - | + |
| D-Aspartic Acid | - | - |
| D-Serine | - | + |
| Troleandomycin | + | + |
| Rifamycin SV | + | + |
| Minocycline | + | - |
| Gelatin | + | + |
| Glycyl-L-Proline | + | + |
| L-Alanine | + | + |
| L-Arginine | - | + |
| L-Aspartic Acid | + | + |
| L-Glutamic Acid | + | + |
| L-Histidine | + | + |
| L-Pyroglutamic Acid | - | + |
| L-Serine | - | + |
| Lincomycin | + | + |
| Guanidine HCl | + | + |
| Niaproof 4 | - | - |
| Pectin | - | - |
| D-Galacturonic Acid | - | - |
| L-Galactonic Acid Lactone | - | - |
| D-Gluconic Acid | + | + |
| D-Glucuronic Acid | - | + |
| Glucuronamid e | - | + |
| Mucic Acid | - | - |
| Quinic Acid | - | - |
| D-Saccharic Acid | - | - |
| Vancomycin | - | - |
| Tetrazolium Violet | - | - |
| Tetrazolium Blue | - | + |
| p-Hydroxy-Phenylacetic Acid | - | - |
| Methyl Pyruvate | + | + |
| D-Lactic Acid Methyl Ester | - | - |
| L-Lactic Acid | + | - |
| Citric Acid | + | + |
| α-Keto-Glutaric Acid | - | - |
| D-Malic Acid | - | - |
| L-Malic Acid | + | + |
| Bromo-Succinic Acid | + | + |
| Nalidixic Acid | + | + |
| Lithium Chloride | + | + |
| Potassium Tellurite | + | + |
| Tween 40 | + | + |
| γ-Amino-Butryric Acid | - | + |
| α-Hydroxy-Butyric Acid | + | + |
| β-Hydroxy-D, L Butyric Acid | - | + |
| α-Keto-Butyric Acid | + | + |
| Acetoacetic Acid | + | + |
| Propionic Acid | + | + |
| Acetic Acid | + | + |
| Formic Acid | - | - |
| Aztreonam | + | + |
| Sodium Butyrate | + | + |
| Sodium Bromate | + | + |
| **Enzyme activity (API ZYM):** |  |  |
| Alkaline phosphatase | + | + |
| Esterase (C4) | + | + |
| Esterase lipase (C8) | + | + |
| Lipase (C14) | + | + |
| Leucine arylamidase | + | + |
| Valine arylamidase | + | + |
| Cystine arylamidase | + | + |
| Trypsin | + | + |
| α-Chymotrypsin | + | + |
| Acid phosphatase | + | + |
| Naphtol-AS-BI-phosphohydrolase | + | + |
| α-Galactosidase | - | + |
| β-Galactosidase | - | + |
| β-Glucuronidase | - | - |
| α-Glucosidase | + | + |
| β-Glucosidase | + | + |
| N-Acetyl-β-glucosaminidase | + | + |
| α-Mannosidase | - | + |
| α-Fucosidase | - | - |
| Fatty Acids (>10%) | anteiso-C_15:0_ and iso-C_16:0_ | iso-C_15:0_, anteiso-C_15:0_ and C_16:0_ |
| Polar Lipid Profile | PE, PME, GL, AL and L | PE |
| Cell Wall Composition | LL-diaminopimelic acid | LL-diaminopimelic acid |
| Whole-cell Hydrolysates | ribose, xylose and arabinose | ribose |
| **Anticancer Activity:** |  |  |
| Activity Against Cell Line HeLa (IC50) | 41.23 µL/mL | - |
| Activity Against Cell Line A549 (IC50) | 45.98 µL/mL | - |
| Activity Against Cell Line ES-2 (IC50) | 36.19 µL/mL | - |
| Activity Against Cell Line MC38 (IC50) | 36.19 µL/mL | - |
| Apoptosis Induction Cell Line HeLa | 10.99% | 7.00% |
| Apoptosis Induction Cell Line A549 | 9.25% | 2.14% |
| Apoptosis Induction Cell Line ES-2 | 13.92% | 3.58% |
| Apoptosis Induction Cell Line MC38 | 10.01% | 2.22% |
| Apoptosis Induction Cell Line NCM460 | 1.96% | 1.90% |
| Cell Cycle Arrest (Phase) | Sub-G1, S and G1 | G1 |

**Note:** Data obtained from this study. +, Positive; -, negative.
