# Supplementary material for: A novel human Streptomyces strain redresses malignancy by inducing apoptosis and regaining gut homeostasis
Source: mSystems. 2026 Sep 23;11(10):e00528-26. doi: 10.1128/msystems.00528-26 (PMC13712931; doi:10.1128/msystems.00528-26)
Supplement: Supplementary file 5 [file msystems.00528-26-s0007.docx]

**Table S5. Fatty acid composition of strain CGMCC 17111 and *S. albidoflavus*** **DSM 40455^T^*.***

| **Fatty acid (%)** | **CGMCC 17111** | **DSM 40455^T^** |
| --- | --- | --- |
| C_8:0_ 3OH | - | 0.43 |
| C_9:0_ | 0.26 | 0.80 |
| C_10:0_ | - | 0.24 |
| C_10:0_ 2OH | - | 0.57 |
| C_10:0_ 3OH | 0.22 | - |
| iso-C_10:0_ | - | 0.88 |
| anteiso-C_11:0_ | 0.18 | - |
| C_12:0_ | - | 1.01 |
| iso-C_12:0_ | 0.21 | - |
| iso-C_12:0_ 3OH | 0.16 | - |
| C_13:0_ 3OH/iso-C_15:1_ H | 0.16 | 0.44 |
| iso-C_13:0_ | 0.40 | 0.54 |
| anteiso-C_13:0_ | 0.51 | 0.67 |
| iso-C_13:0_ 3OH | 0.32 | 0.91 |
| iso-C_14:0_ | 9.93 | 2.53 |
| anteiso-C_14:0_ | 0.53 | - |
| iso-C_15:0_ | 5.55 | 13.73 |
| iso-C_15:0_ 3OH | - | 0.16 |
| anteiso-C_15:0_ | 21.59 | 28.77 |
| iso-C_15:1_ F | 0.20 | 0.70 |
| anteiso-C_15:1_ A | 0.04 | 0.18 |
| C_16:0_ | 8.59 | 14.25 |
| iso-C_16:0_ | 27.51 | 5.35 |
| iso-C_16:0_ 3OH | 0.25 | - |
| anteiso-C_16:0_ | 0.23 | - |
| C_16:0_ 2OH | 0.11 | - |
| iso-C_16:1_ I/C_14:0_ 3OH | - | 0.51 |
| iso-C_16:1_ H | 1.12 | - |
| C_16:1_ ω7c/C_16:1_ ω6c | 2.00 | 0.33 |
| C_17:0_ | 0.85 | 0.53 |
| iso-C_17:0_ | 2.28 | 8.32 |
| iso-C_17:0_ 3OH | 0.16 | - |
| anteiso-C_17:0_ | 9.26 | 9.06 |
| C_17:0_ cyclo | 2.51 | - |
| C_17:0_ 10-methyl | 0.18 | - |
| C_17:0_ 2OH | 0.19 | 0.21 |
| C_17:0_ 3OH | 0.10 | - |
| iso-C_17:1_ω9c | 0.63 | - |
| iso-C_17:1_ I/anteiso-C_17:1_ B | 0.09 | - |
| iso-C_17:1_ω5c | - | 0.25 |
| anteiso-C_17:1_ω9c | 0.84 | - |
| C_17:1_ω8c | 0.30 | - |
| C_18:0_ | 0.56 | 3.01 |
| iso-C_18:0_ | 0.46 | 0.46 |
| C_18:2_ω6,9c/ante-C_18:0_ | 0.13 | 0.32 |
| C_18:1_ω7c | 0.16 | 0.36 |
| C_18:1_ω9c | 0.39 | 0.65 |
| iso-C_19:0_ | - | 0.43 |
| anteiso-C_19:0_ | - | 0.41 |
| C_20:0_ | - | 0.29 |

**Note: –, composition absent.**
